# An RNA Thermometer Activity of the West Nile Virus Genomic 3′-Terminal Stem-Loop Element Modulates Viral Replication Efficiency during Host Switching

**DOI:** 10.3390/v12010104

**Published:** 2020-01-15

**Authors:** Alexandra Meyer, Marie Freier, Tobias Schmidt, Katja Rostowski, Juliane Zwoch, Hauke Lilie, Sven-Erik Behrens, Susann Friedrich

**Affiliations:** Institute of Biochemistry and Biotechnology, Martin Luther University Halle-Wittenberg; 06108 Halle (Saale), Germany; alexandra.meyer@biochemtech.uni-halle.de (A.M.); marie.freier@gmx.de (M.F.); T.Schmidt@beatson.gla.ac.uk (T.S.); katja.rostowski@biochemtech.uni-halle.de (K.R.); juliane.zwoch@gmx.de (J.Z.); hauke.lilie@biochemtech.uni-halle.de (H.L.)

**Keywords:** West Nile virus, host switching, RNA thermometer, host factor, RNA chaperone, RNA annealer, RNA remodeling, RNA structure, Flavivirus, RNA replication

## Abstract

The 3′-terminal stem-loop (3′SL) of the RNA genome of the flavivirus West Nile (WNV) harbors, in its stem, one of the sequence elements that are required for genome cyclization. As cyclization is a prerequisite for the initiation of viral replication, the 3′SL was proposed to act as a replication silencer. The lower part of the 3′SL is metastable and confers a structural flexibility that may regulate the switch from the linear to the circular conformation of the viral RNA. In the human system, we previously demonstrated that a cellular RNA-binding protein, AUF1 p45, destabilizes the 3′SL, exposes the cyclization sequence, and thus promotes flaviviral genome cyclization and RNA replication. By investigating mutant RNAs with increased 3′SL stabilities, we showed the specific conformation of the metastable element to be a critical determinant of the helix-destabilizing RNA chaperone activity of AUF1 p45 and of the precision and efficiency of the AUF1 p45-supported initiation of RNA replication. Studies of stability-increasing mutant WNV replicons in human and mosquito cells revealed that the cultivation temperature considerably affected the replication efficiencies of the viral RNA variants and demonstrated the silencing effect of the 3′SL to be temperature dependent. Furthermore, we identified and characterized mosquito proteins displaying similar activities as AUF1 p45. However, as the RNA remodeling activities of the mosquito proteins were found to be considerably lower than those of the human protein, a potential cell protein-mediated destabilization of the 3′SL was suggested to be less efficient in mosquito cells. In summary, our data support a model in which the 3′SL acts as an RNA thermometer that modulates flavivirus replication during host switching.

## 1. Introduction

Along with other human pathogens such as the dengue virus (DENV), Zika virus (ZIKV), and Japanese encephalitis virus (JEV), the West Nile virus (WNV) belongs to the genus *Flavivirus* within the virus family *Flaviviridae*. The WNV, which cycles between mosquitos and birds, is currently the arbovirus with the widest geographic distribution. Humans and horses are incidental hosts and they do not play a role in the transmission cycle [1]. The interactions among flaviviruses, invertebrate vectors, and vertebrate hosts are complex, and increasing temperatures, such as could occur during climate change, affect virus transmission by mosquitos [2].

**Figure 1:**
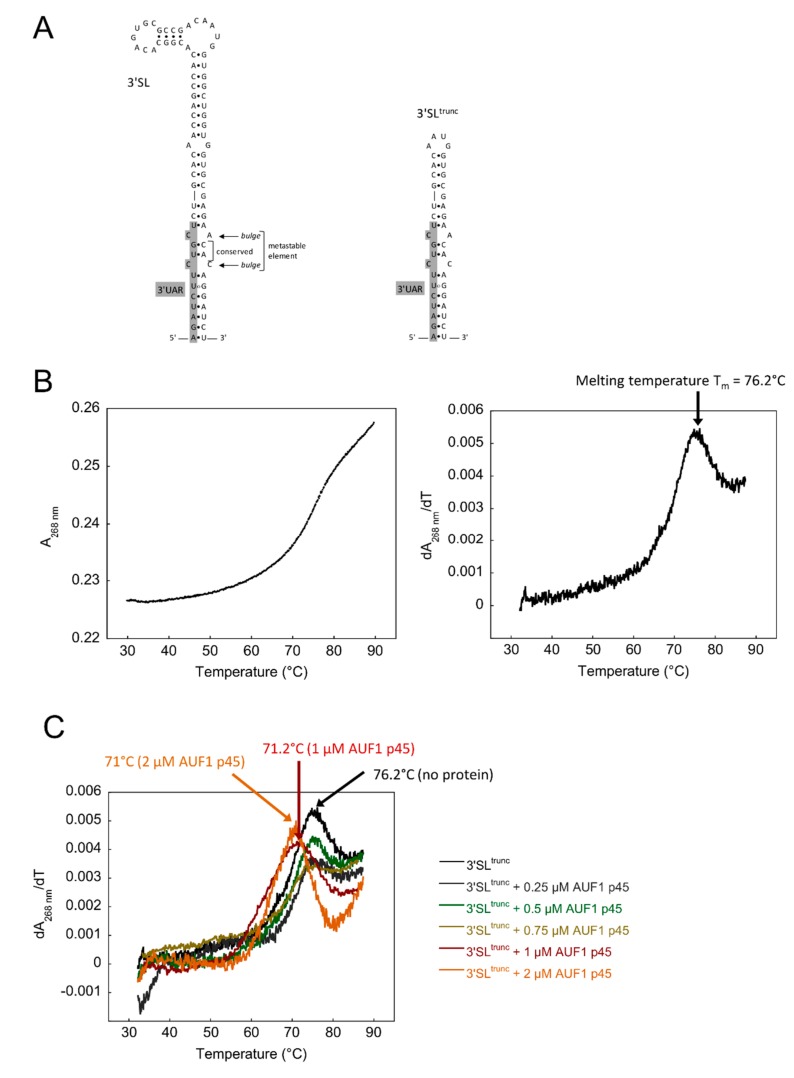
AUF1 p45 increases the flexibility of the WNV 3′SL. (**A**) Sequence and secondary structure of the full-length WNV 3′SL (left) and a truncated 3′SL (3′SL^trunc^) that was used for the thermal denaturation experiments (right). The 3′UAR cyclization sequence is shown in grey. The metastable element within the bottom part of the 3′SL consists of two conserved base pairs and is flanked by two bulges. (**B**) (left) Thermal denaturation of the 3′SL^trunc^ RNA. The denaturation was recorded in the temperature range from 30 to 90 °C. (right) First derivative of the trace shown on the left to determine the melting temperature T_M_ (arrow). (**C**) First derivatives of thermal denaturation experiments of the 3′SL^trunc^ RNA in the absence and presence of increasing concentrations of AUF1 p45. Melting temperatures are indicated with arrows.

The flaviviral genome is an approximately 11 kb long single-stranded RNA of positive polarity containing a single open reading frame (ORF), which encodes three structural (core, membrane, and envelope) and seven nonstructural proteins (NS1, NS2A, NS2B NS3 NS4A, NS4B, and NS5) [3]. The ORF is flanked by highly structured 5′- and 3′-untranslated regions (UTRs), providing a set of cis-acting elements that support a switch in the RNA structure of the genome from a linear to a 5′-3′ cyclized form, which, in turn, is an essential prerequisite for the initiation of RNA synthesis [4]. Genome cyclization enables the NS5 polymerase, which specifically interacts with the SLA structure at the 5′-end of the genome, to be transferred to the 3′-end in order to initiate the first step of RNA replication, the synthesis of negative-strand RNA replication intermediates [5,6,7]. Genome cyclization requires structural rearrangements of the viral RNA, which ultimately enable the hybridization of complementary cyclization sequences, termed CS, UAR, and DAR sequences at the 5′ and 3′ termini (reviewed in [8]). Importantly, in the linear form of the flaviviral genome, the 3′UAR cyclization sequence is part of the 5′-side of the stem structure of the 3′-terminal stem-loop (3′SL). Accordingly, to enable genome cyclization, the 3′SL has to be denatured to allow hybridization with the complementary 5′UAR sequence within the 5′UTR [9,10]. The lower part of the 3′SL contains a metastable element, which consists of two conserved base pairs that are flanked by two symmetrical bulges (Figure 1A). Previous studies have suggested that the metastable element participates in regulating the RNA switch from a linear to a circular conformation as follows: (1) Mutations that increase the stability of the 3′SL have negative effects on WNV RNA synthesis and propagation [11] and (2) a structural change of the DENV 3′SL is required for the initiation of viral negative-strand RNA synthesis in vitro [12].

In previous work, we have shown that the human RNA-binding protein AUF1 p45 modifies flaviviral RNA structures and that this involves two RNA remodeling activities of the protein, an RNA chaperone and an RNA annealing activity [13,14,15]. Specifically, via its RNA chaperone activity, AUF1 p45 destabilizes the 3′SL, thus, exposing the 3′UAR sequence, which is then available to hybridize with the complementary 5′UAR sequence. The RNA annealing activity of AUF1 p45 was demonstrated to accelerate the hybridization of complementary cyclization sequences. Thus, AUF1 p45 was found to stimulate 5′-3′-interactions of flaviviral RNAs and, with this, the first step of the viral replication process, the synthesis of negative-strand replication intermediates. In this context we could also show that the RGG/RG motif of AUF1 p45 is a key modulator of the protein’s RNA remodeling activities [16].

In this study, we present a set of complementary experimental data obtained in the human and mosquito systems, which suggest a molecular model in which the WNV 3′SL acts as an RNA thermometer during host switching.

## 2. Materials and Methods

### 2.1. Thermal Denaturation of RNA

Thermal denaturation was done as described in [17] with minor modifications. Briefly, 250 nM of the wild-type and mutant 3′SL^trunc^ RNAs were incubated with the indicated concentrations of AUF1 p45 in 50 mM Hepes/NaOH, 100 mM KCl, and 5 mM MgCl_2_, at pH 8.0. Thermal denaturation was monitored in the range from 30 to 90 °C at 0.4 K min^−1^ by recording the absorbance change at a constant wavelength of 268 nm on a JASCO-V550 equipped with temperature control unit ETC-505T. Absorbance changes relative to the signal at 30 °C were plotted against the temperature and transition curves were analyzed semi-quantitatively by calculating the melting points (T_M_) from the 1st derivative of each curve.

### 2.2. Plasmids Encoding WNV Replicons

The plasmid encoding a subgenomic WNV RNA (pWNVsg) has been described in [13]. The pWNVsg derivatives with mutant 3′SL Mut1, 3′SL Mut2, and 3′SL Mut3 were obtained by PCR-based site-directed mutagenesis using the primers provided in Supplementary Table S1. The Renilla luciferase encoding WNV replicon construct (pWNVRluc) has been described in [13]. To obtain replicon encoding cDNAs with mutations in the 3′SL, the SacII-XbaI fragment of pWNVsg with the corresponding mutation was cloned into pWNVRluc.

### 2.3. Plasmids Encoding SUMO-Fusion Proteins

The pETSUMO expression system was used for the expression of recombinant proteins in *Escherichia coli*. The plasmids encoding human AUF1 p45 and WNV NS5 are described elsewhere in [13]. The cDNAs encoding the mosquito squid proteins p30 and p32 were generated by reverse-transcription-PCR of total mRNA isolated from C6/36 cells (primer sequences are supplied in Supplementary Table S1) and cloned into the pETSUMO expression vector.

### 2.4. Plasmids Encoding FLAG-Fusion Proteins

The cDNAs encoding the squid proteins p30 and p32 were amplified from the pETSUMO expression vectors (primer sequences are supplied in Supplementary Table S1) and cloned into pSinRep5. The FLAG-coding sequence was introduced via the forward PCR primer such that the corresponding proteins contained the FLAG-tag fused to their N-terminus.

### 2.5. Expression and Purification of WNV NS5 and AUF1 p45

Expression and purification of WNV NS5 and AUF1 p45 were performed as described in [13].

### 2.6. Expression and Purification of Aedes albopictus p30 and p32

Mosquito squid proteins p30 and p32 were purified from the soluble fraction of *Escherichia coli* BL21-CodonPlus^®^ (DE3)-RP cells using nickel-agarose affinity chromatography and, after cleavage with SUMO-protease by heparin-sepharose affinity chromatography followed by gel-filtration chromatography (HiLoad^TM^ 16/60 Superdex 75^TM^, GE Healthcare, Amersham, UK). UV absorption spectra were measured using a JASCO V-550 spectrometer. The protein concentration was determined by measuring the absorbance at 280 nm using ε_280_ = 24410 M^−1^ cm^−1^ for p30 and ε_280_ = 44350 M^−1^ cm^−1^ for p32. The proteins were stored at −80 °C in 20 mM Tris/HCl, pH 7.6, 150 mM KCl, and 1 mM Tris (2-carboxyethyl) phosphine (TCEP).

### 2.7. Expression and Purification of FLAG-p30 and -p32 Fusion Proteins

The Sindbis expression system (Thermo Fisher Scientific, Waltham, MA, USA) was used for the transient expression of mosquito squid proteins p30 and p32 in C6/36 cells. Recombinant Sindbis replicon RNA (3 µg) encoding the FLAG fusion proteins FLAG-30 or FLAG-p32 was transfected into approximately 2.4 × 10^7^ C6/36 cells by electroporation (Bio-Rad Gene Pulser, 1 pulse without controller at 0.3 kV and 300 µF, resistance ∞, and 4 mm gap cuvettes). After 24 h, the cells were harvested, and the cytoplasmic extracts were subjected to treatment with anti-FLAG M2 affinity gel (Sigma, St. Louis, MO, USA) in the presence of 0.1 mg/mL RNase A, at 4 °C overnight. The FLAG fusion proteins were eluted with 3XFLAG peptide (Sigma) at a final concentration of 500 ng/µl. The amount of eluted protein was estimated by a dilution series of FLAG-tagged AUF1 p45 with a known concentration, which was prepared as in [14].

### 2.8. In Vitro Transcription

All replicon encoding plasmids were transcribed by runoff in vitro transcription (standard protocol) with T7 or SP6 RNA polymerase (Agilent Technologies, Waldbronn, Germany; Thermo Fisher Scientific, Waltham, MA, USA) in the presence of m^7^GpppG cap analogue (Jena Bioscience, Jena, Germany) at a 1.6:1 molar ratio of m^7^GpppG/GTP. The WNV sgRNAs were transcribed with T7 RNA polymerase from PCR products that were generated from the respective plasmids (primer sequences are supplied in Supplementary Table S1).

### 2.9. Cells, Culturing, and Transfection Conditions, Luciferase Assay

Human hepatoma cells (Huh7) were cultured in Dulbecco’s modified Eagle’s medium (DMEM; Gibco) supplemented with 10% fetal calf serum (FCS; PAN-Biotech, Aidenbach, Germany), 1% penicillin/streptomycin, 0.1% D-biotin, and 0.1% hypoxanthine. Approximately 0.4 × 10^6^ Huh7 cells were transfected with 1 µg WNVRluc replicon RNA using the Bio-Rad Gene Pulser (1 pulse without controller at 0.2 kV and 950 µF, resistance ∞, and 4 mm gap cuvettes). The C6/36 cells (ATCC) and U4.4 cells (kindly provided by Ronald van Rij) were cultured in Leibovitz’s L-15 medium supplemented with 10% fetal calf serum (FCS; PAN-Biotech), 1% penicillin/streptomycin, 1% NEAA (non-essential amino acids), 2% tryptose phosphate. Approximately 2.4 × 10^7^ C6/36 cells were transfected with 3 µg WNVRluc replicon RNA using the Bio-Rad Gene Pulser (1 pulse without controller at 0.3 kV and 300 µF, resistance ∞, and 4 mm gap cuvettes). A luciferase assay kit was used to quantify the activity of the replicon encoded Renilla luciferase (Promega, Walldorf, Germany).

### 2.10. Replicase Assay

The assay was performed in a total volume of 40 μL in buffer containing 50 mM Hepes/NaOH, 10 mM KCl, 5 mM MgCl_2_, 0.5 mM MnCl_2_, and 1 mM dithiothreitol, at pH 8.0. It contained 500 μM (each) ATP, GTP, and UTP, 0.1 µM CTP, 10 μCi [α-^32^P] CTP, 10 nM of template RNA, and 15 nM of the recombinant, purified NS5. Supplementation was performed such that 200 nM AUF1 p45 was preincubated under the assay conditions with the template RNA, and NS5 was added subsequently. The reaction was carried out for 60 min at 37 °C and stopped by phenol/chloroform extraction and ethanol precipitation of the RNA. The RNA products were analyzed by electrophoresis on a 5% denaturing polyacrylamide gel (7 M urea), visualized by phosphor imaging and quantified by ImageQuant Software (GE Healthcare).

### 2.11. Western Blotting and Antibodies

To detect *A. albopictus* p30 and p32 in Western blots, a rabbit polyclonal antiserum was applied that was raised against the recombinant, purified full-length p32 protein (Eurogentec, Liège, Belgium). Secondary antibodies were purchased from LI-COR Biosciences (Bad Homburg, Germany). Western blots were performed using standard protocols and by following the instructions of the manufacturers.

### 2.12. Measurement of RNA-Binding Constants

Mosquito squid proteins p30 or p32 were added to 5′-FAM-EX-labeled RNA (5 to 25 nM) in assay buffer (50 mM Hepes/NaOH, pH 8.0, 100 mM KCl, and 5 mM MgCl_2_). Fluorescence changes were monitored using a Fluoromax-4 spectrofluorometer (Jobin Yvon, Bensheim, Germany) at 22 °C. The signal amplitudes of the 5′-FAM-EX-labeled RNAs (Supplementary Table S2) were measured (excitation at 491 nm and emission at 515 nm) and corrected for the volume change. Fluorescence intensities relative to the starting fluorescence were plotted against the protein concentration. Fitting the binding isotherms to a single-site binding model according to Equation (1) [16] with KaleidaGraph 4.5 (Synergy Software, Reading, PA, USA) yielded the *K*_D_ values of the interaction of the protein and the labeled RNA.

(1) $$\Delta F=1+\gamma \cdot \left[\frac{(b+c+K_{D})-\sqrt{{(b+c+K_{D})}^{2}-4\cdot b\cdot c}}{2\cdot b}\right]$$

where ΔF is the relative change in fluorescence intensity, γ is the signal amplitude, b is the total concentration of the RNA, c is the total concentration of the protein, and *K*_D_ is the dissociation constant.

### 2.13. In Vitro Methylation Assay

Either 1 pmol of FLAG-p30 or FLAG-p32 (purified from C6/36 cells), or 1 pmol p30 or p32 (purified from *E. coli*) and 5 pmol of protein arginine methyltransferase 1 (PRMT1, purified according to [14]) were incubated in a reaction mixture that contained 50 mM Hepes/NaOH, pH 8.0, 10 mM KCl, 5 mM MgCl_2_, 0.2 mg/mL bovine serum albumin, 0.5 mM dithiothreitol, and 40 µM [S-**^14^**C]adenosylmethionine (60 mCi/mmol, GE Healthcare, Amersham, UK). The reaction was performed at 30 °C for 2 h and 13 pmol AUF1 p45 (purified from *E. coli*) served as a positive control. The degree of methylation was analyzed by SDS-PAGE and phosphor imaging.

### 2.14. Fluorescence-Based RNA-RNA Interaction Assay

The assay was performed as previously described in [13]. Briefly, the purified, recombinant proteins were added to 10 nM of 5′-Cy5-labeled 3′SL^trunc^-RNA (purchased from IBA, Göttingen, Germany; Supplementary Table S2) at different concentrations in assay buffer (50 mM Hepes/NaOH, pH 8.0, 100 mM KCl, and 5 mM MgCl_2_). Then, 100 nM of non-labeled 5′UAR RNA was added and readings were taken for another 400 s. Changes in the fluorescence signals were monitored in a Fluoromax-4 spectrofluorometer (Jobin Yvon, Bensheim, Germany) at 22 °C with the following parameters: excitation at 643 nm and emission at 667 nm. Fluorescence intensities relative to the starting fluorescence were plotted against the time and fitted by KaleidaGraph 4.5 (Synergy, Reading, PA, USA) to first-order reaction when protein was omitted (Equation (2)) and second-order reaction when protein was included (Equation (3)) yielding the corresponding rate constants *k*_obs_.

ΔF = F_offset_ + F_max_∙[1 − exp(−*k*_obs_∙t)] (2)

∆F = F_offset_ + F_max_∙[1 − 1/(*k*_obs_∙t + 1)] (3)

where ΔF is the total change of relative fluorescence amplitude, F_offset_ is the fluorescence intensity at the start point of the reaction, F_max_ is the maximum signal amplitude, *k*_obs_ is the observed rate constant, and *t* is time.

### 2.15. FRET-Based RNA Annealing of Complementary Cyclization Sequences

The assay was performed as previously described in [14]. Briefly, the fluorescent oligonucleotides (Supplementary Table S2) were purchased from IBA (Göttingen, Germany; Supplementary Table S2). The purified, recombinant proteins were added at different concentrations to 10 nM of 5′-Cy3-labeled 3′CS-RNA in assay buffer (50 mM Hepes/NaOH, pH 8.0, 100 mM KCl, and 5 mM MgCl_2_). Then, 10 nM of 5′-Cy5-labeled 5′CS-RNA was added, and readings were taken for another 400 s. Changes in the fluorescence signals were monitored in a Fluoromax-4 spectrofluorometer (Jobin Yvon, Bensheim, Germany) at 22 °C. The Cy3 fluorophore was excited at 535 nm wavelength and readings were taken at the Cy5 emission wavelength 680 nm. Fluorescence intensities relative to the starting fluorescence were plotted against the time and fitted by KaleidaGraph 4.5 (Synergy software, Reading, PA, USA) to a second-order reaction (Equation (3), see above) yielding the corresponding rate constants *k*_obs_.

### 2.16. Analytical Ultracentrifugation

Sedimentation equilibrium measurements were performed as previously described in [14]. Analysis was carried out at a protein concentration of 12 µM (mosquito p30) and 7 µM (mosquito p32) in 20 mM Tris/HCl, pH 7.6, 150 mM KCl, and 1 mM TCEP at 20 °C.

### 2.17. Circular Dichroism

Measurements of dichroic properties of mosquito proteins p30 and p32 were performed using a JASCO J-810 spectropolarimeter. Far-UV circular dichroism spectra were recorded at a protein concentration of 2 µM in 20 mM Tris/HCl, pH 7.6, 150 mM KCl, and 1 mM TCEP at 22 °C using cuvettes with optical path lengths of 1 mm. Acquired protein spectra were corrected for buffer contribution and smoothed using the Spectra Manager I software (JASCO). The data were converted to mean residue ellipticity Θ_MRW_.

## 3. Results

### 3.1. Increased Stability of the WNV 3′SL Negatively Affects the RNA Chaperone Activity of AUF1 p45

In previous studies, we have shown that AUF1 p45 destabilizes the 3′SL, thus, exposing the 3′UAR cyclization sequence and promoting cyclization and RNA replication [13,15]. To gain more insight into AUF1 p45-mediated destabilization of the WNV 3′SL, we performed thermal denaturation studies. We applied a truncated version of the 3′SL (3′SL^trunc^), which harbors the 3′UAR cyclization sequence, as well as the metastable element with the two flanking bulges (Figure 1A) and analyzed the thermal melting behavior of this RNA in the absence and presence of AUF1 p45. The 3′SL^trunc^ RNA alone exhibited a transition midpoint (melting temperature) at 76.2 °C calculated from the first derivative of the transition curve (Figure 1B). In the presence of increasing concentrations of AUF1 p45 the transition midpoint shifted towards lower temperatures (Figure 1C) indicating a lower thermal stability of the 3′SL^trunc^ RNA. These observations confirm the ability of AUF1 p45 to destabilize flaviviral stem-loop RNAs.

**Figure 2:**
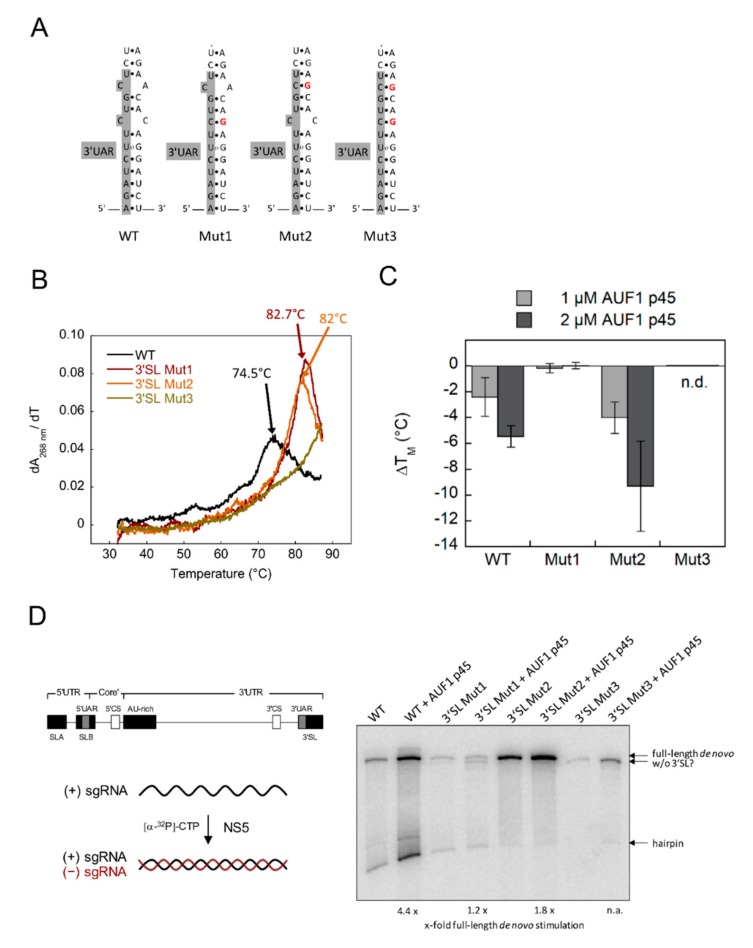
Increasing stability of the WNV 3′SL correlates with decreasing RNA chaperone activity of AUF1 p45. (**A**) Sequence and secondary structure of the bottom part of the WNV 3′SL and mutants. Introduced mutations are shown in red. The 3′UAR cyclization sequence is shown in grey. (**B**) First derivatives of thermal denaturation experiments of the 3′SL^trunc^ wild-type and mutant RNAs. Melting temperatures are indicated with arrows. (**C**) Change of melting temperatures of thermal denaturation experiments of the 3′SL^trunc^ wild-type and mutant RNAs in the presence of AUF1 p45. n.d., not definable. Average results and standard deviations (*n* = 3) are shown. (**D**) Schematic drawing of the WNV sgRNA and experimental outline of the replicase assay. The RNA consists of the 5′- and 3′UTR and a part of the core coding sequence. It contains the 5′SLA, the 3′SL, all cyclization elements, and the AU-rich element in the upstream portion of the 3′UTR (left). WNV sgRNAs that contained the wild-type or mutant 3′SL were tested in the replicase assay in the absence or presence of AUF1 p45. The products were analyzed by denaturing PAGE and phosphor imaging. One representative experiment is shown (right). Levels of stimulation of full-length de novo product relative to the control (absence of AUF1 p45) are given below. n.a., not applicable.

Previous data suggested that the metastable element of the 3′SL confers a conformational flexibility that regulates long-range 5′-3′ RNA–RNA interactions within the viral genome [11]. Considering the important role that AUF1 p45 plays in this process, we hypothesized that the structural flexibility of the 3′SL is necessary for AUF1′s helix-destabilizing RNA chaperone activity. Therefore, we introduced mutations into the metastable element, which should increase the stability of the 3′SL. We generated three mutant 3′SL^trunc^ RNAs in order to close either the lower bulge (3′SL Mut1), the upper bulge (3′SL Mut2), or both bulges (3′SL Mut3) (Figure 2A). To maintain 5′-3′UAR complementarity, we introduced the mutations within the 3′-side of the 3′SL. First, we tested whether the mutant 3′SL^trunc^ RNAs actually showed increased stability by analyzing their thermal melting behavior. The 3′SL Mut1 and Mut2 RNAs exhibited transition midpoints of 82.7 °C and 82 °C, respectively, while the 3′SL Mut3 was too stable to determine accurately a melting temperature (Figure 2B). Next, we tested the activity of AUF1 p45 with these RNAs. Interestingly, with 3′SL Mut1 we did not observe a change in the melting temperature in the presence of AUF1 p45. In contrast, 3′SL Mut2 was destabilized by AUF1 p45 in a similar way to the situation with the wild-type 3′SL. With the most stable mutant RNA, 3′SL Mut3, the melting behavior was unchanged in the absence or presence of AUF1 p45 (Figure 2C). These data indicate that a specific conformational flexibility of the metastable element of the WNV 3′SL RNA is essential for an optimal RNA chaperone activity of AUF1 p45. While closing of the lower bulge and both bulges is detrimental to the protein’s activity, closing of the upper bulge is tolerated.

Next, we tested how the mutations affected AUF1 p45-supported WNV negative-strand RNA synthesis. For this purpose, we performed a previously established in vitro replicase assay using the purified WNV RNA-dependent RNA polymerase (RdRp) NS5 and, as a template, a subgenomic RNA (sgRNA). The assay essentially generates two RNA products, namely a negative-strand RNA product, which results from de novo initiation of RNA polymerization on the template, and a hairpin product, which is synthesized by priming at the 3′-OH of the template and turn-around copy-back polymerization by the NS5 RdRp [13]. De novo RNA synthesis by NS5 itself was clearly affected by the mutations in the 3′SL. With the 3′SL Mut1 sgRNA we observed an evident decrease in the amount of the full-length de novo RNA product as compared with the wild-type control (Figure 2D). With the 3′SL Mut2 sgRNA template, the ratio of the NS5-generated de novo product to the hairpin product was changed in favor of the de novo synthesized RNA, suggesting that this mutation inhibited the capability of NS5 to prime RNA synthesis at the template’s 3′-end. Interestingly, when using the 3′SL Mut3 as a template, NS5 was unable to produce the full-length RNA, and only resulted in a shorter RNA product. This indicates that with the most stable variant, 3′SL Mut3, NS5 is incapable of de novo initiation of RNA synthesis at the very 3′-end, but rather initiates upstream of the 3′SL. As observed previously [13], AUF1 p45 was able to promote negative-strand RNA synthesis by the polymerase NS5 using the wild-type sgRNA as a template (Figure 2D). Remarkably, with the sgRNA 3′SL Mut1 we did not observe an increase in the amount of the full-length de novo RNA product in the presence of AUF1 p45, but rather detected an additional shorter product was made (Figure 2D). With the 3′SL Mut2 sgRNA template, de novo negative-strand RNA synthesis was stimulated in the presence of AUF1 p45, but this was less efficient than with the wild-type sgRNA. When using the 3′SL Mut3 sgRNA as a template, we observed an increase in the amount of the shorter RNA product in the presence of AUF1 p45, but no full-length de novo RNA product was observed (Figure 2D).

Together, the data imply that (1) an increased stability of the WNV 3′SL (at least in the case of Mut1 and Mut3) prevents precise initiation of RNA synthesis by NS5 at the 3′-end of the viral RNA, and that (2) the host factor AUF1 p45 stimulates full-length de novo RNA synthesis only if a certain structural flexibility of the 3′SL is provided.

### 3.2. Increased Stability of the 3′SL Affects WNV RNA Replication Differently in Human and Mosquito Cells

**Figure 3:**
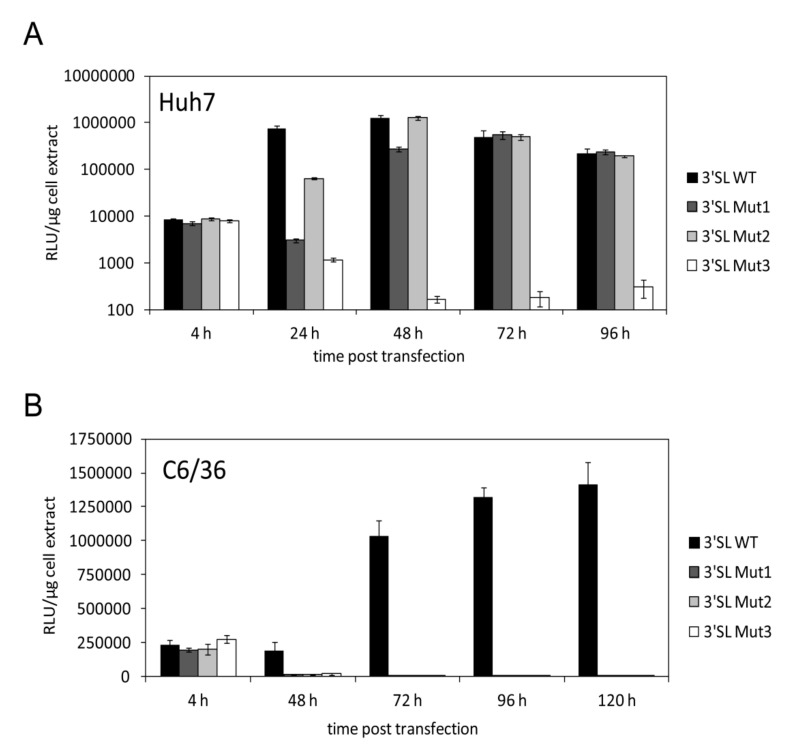
Altering the stability of the WNV 3′SL affects RNA replication differently in human and mosquito cells. (**A**) Huh7 cells were transfected with wild-type and mutant WNVRluc replicon RNAs, cultivated at 37 °C and analyzed for luciferase reporter activity at the indicated time points post transfection. Results from one representative experiment performed in triplicate are shown and error bars reflect standard deviations. (**B**) Same as in (A) except that C6/36 cells were used and cultivated at 28 °C.

To assess the role of the stability of the 3′SL on WNV replication in different host cells, we introduced each mutation individually into a WNV luciferase reporter replicon [13] that enables focused studies of the translation and replication processes of the viral RNA. The luciferase activity at 4 h post transfection (p.t.) was used as an indicator for translation of the input RNA, while later time points represented RNA replication. While in the human cell line Huh7, the translation levels of all mutant replicons were similar to those with the wild-type replicon, their replication efficiencies were differentially affected (Figure 3A). The 3′SL Mut1 and 3′SL Mut2 replicons showed a delayed RNA replication phenotype, with 3′SL Mut1 reaching wild-type levels at 72 h p.t. and 3′SL Mut2 at 48 h p.t. The replicon containing the 3′SL Mut3 mutation was replication-deficient (Figure 3A). Interestingly, the replication efficiencies of the replicon RNAs inversely correlated with the stability of the 3′SL (see Figure 2B).

We observed a different picture in the mosquito cell line C6/36. While the translation levels of all mutant replicons were again similar to those of the wild-type replicon, all mutant replicons turned out to be replication deficient (Figure 3B). Additionally, we observed different replication maxima for the wild-type replicon. While maximum replication was detected at 48 h p.t. in Huh7 cells, replication in mosquito cells was highest at 96 to 120 h p.t. These results show that in mosquito cells WNV RNA replication is preceded by a longer lag phase as compared with human cells. In addition, the results indicate that, at least under normal growth conditions, the functional impact of increased 3′SL stability on WNV replication differs in human and mosquito cells.

### 3.3. High Temperature Renders Replication-Incompetent Mutant WNV Replicons Replication-Competent in Mosquito Cells

**Figure 4:**
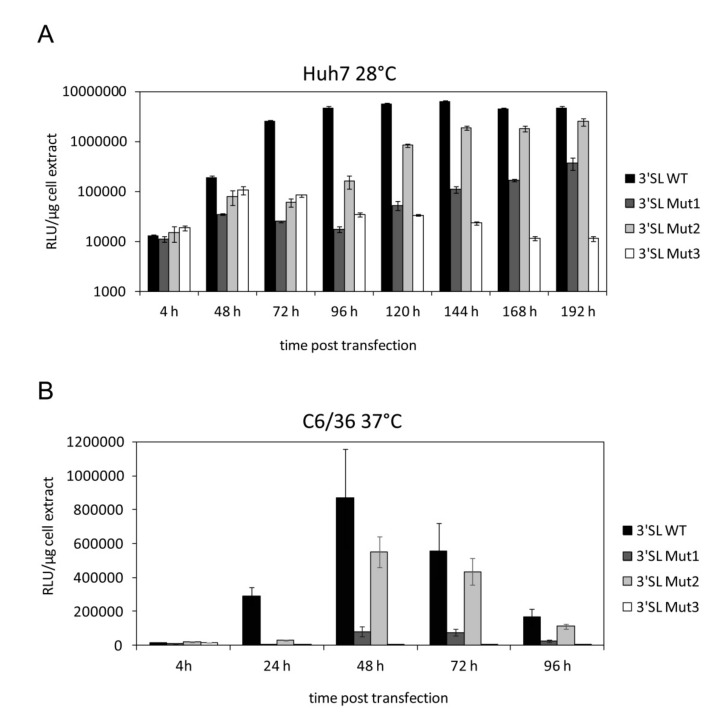
The cultivation temperature determines the replication rates of WNVRluc replicons. (**A**) Huh7 cells were transfected with wild-type and mutant WNVRluc replicon RNAs, subsequently cultivated at 28 °C and analyzed for luciferase reporter activity at the indicated time points post transfection. Results from one representative experiment performed in triplicate are shown and error bars reflect standard deviations. (**B**) Same as in (A) except that C6/36 cells were used and cultivated at 37 °C after transfection.

Next, we asked whether the replication-defective phenotypes of the 3′SL mutant replicons in human and mosquito cells were simply caused by different cultivation temperatures. Therefore, we swapped the cultivation temperatures and performed the Huh7 experiment at 28 °C and the C6/36 experiment at 37 °C. Interestingly, the lag phase preceding replication of the WNV replicon was considerably longer when the Huh7 cells were cultivated at 28 °C (see Supplementary Figure S1). With the wild-type replicon, this resulted in a prolonged translation phase and a late replication maximum of 144 h p.t. (Figure 4A).

In comparison to the wild-type replicon, the 3′SL Mut1 and 3′SL Mut2 replicons showed again a delayed RNA replication phenotype. However, in contrast to the situation at 37 °C, the delay of replication at 28 °C was more pronounced and the mutant replicons barely replicated to wild-type levels. The replicon containing the 3′SL Mut3 mutation was replication-deficient at 28 °C, as it was at 37 °C (Figure 4A).

The replication of the wild-type replicon RNA in the C6/36 cells that were cultured at 37 °C after transfection increased significantly already after 24 h and, accordingly, showed no lag phase (Figure 4B). Therefore, an elevated temperature leads to an acceleration of the replication of WNV RNA in C6/36 cells. Strikingly, the 3′SL Mut1 and 3′SL Mut2 replicons, which were replication-deficient at 28 °C, replicated at a cultivation temperature of 37 °C, although with a delayed phenotype and with lower efficiency as compared with the wild-type. The replicon containing the 3′SL Mut3 mutation was replication-deficient at 37 °C, as it was at 28 °C (Figure 4B). These results indicate that the cultivation temperature decisively determines the replication efficiency of the viral RNA in human and mosquito cells. Thus, the longer lag phase of the replication process of the WNV replicon in mosquito cells was indicated not to be cell specific but rather to depend on the cultivation temperature.

### 3.4. Mosquito Cells Encode Proteins Showing Large Homologies to AUF1

**Figure 5:**
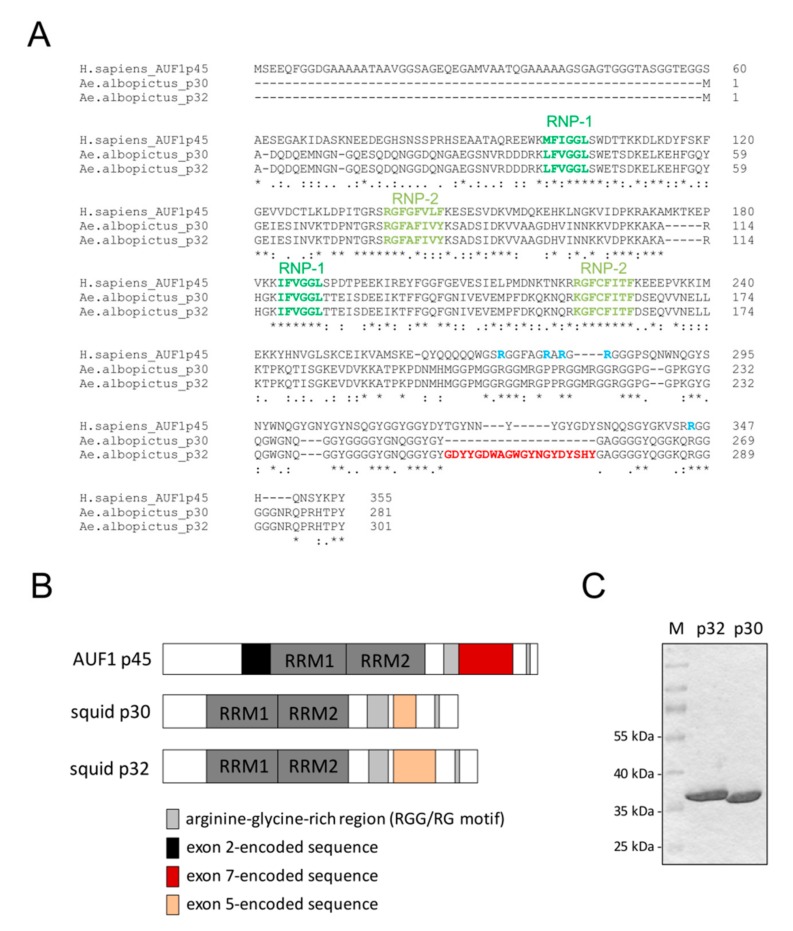
Mosquito cells encode two AUF1-homologous proteins. (**A**) Alignment of human AUF1 p45 and *A. albopictus* proteins p30 and p32. Conserved RNP-1 (light green) and RNP-2 (green) sequences of the RNA recognition motifs (RRM) are highlighted. The arginine residues of the RGG/RG motif of AUF1 p45 that were found to be dimethylated in human cells are highlighted in blue. The alternatively spliced sequence that is absent in p30 but present in p32 is shown in red. (**B**) Domain organization of human AUF1 p45 and mosquito squid proteins p30 and p32. (**C**) AUF1-homologous proteins p30 and p32 were produced in, and purified from, *E. coli*. About 4 μg of protein were analyzed on a Coomassie-stained SDS-gel in parallel with a molecular weight marker (M).

Similar to the situation with human cells, it is likely that RNA-binding proteins within the mosquito host modulate viral RNA conformations. Therefore, we wondered if RNA-binding proteins with activities similar to AUF1 existed in mosquito cells. A sequence similarity search based on AUF1 p45 predicted four proteins in *Aedes albopictus* (the mosquito species from which the cell line C6/36 was derived) containing two RNA recognition motifs (RRMs), an arginine-glycine-rich region (RGG), and a tyrosine-glycine-rich region (YGG), and therefore appear to be very similar to AUF1 (Figure 5A,B). From total RNA of C6/36 cells, we cloned the cDNAs encoding two proteins, which we named squid p30 and squid p32 due to shared homologies to the Drosophila squid proteins. The smaller p30 differs from p32 by a 20 amino acid indel and is generated by an alternative splice site selection within exon 5 (Figure 5B). Further analysis of the genomic location and the amplified cDNA revealed that in C6/36 cells the proteins actually derive from two gene loci (Supplementary Figure S2).

### 3.5. Characterization of the Mosquito Proteins Squid p30 and p32

**Figure 6:**
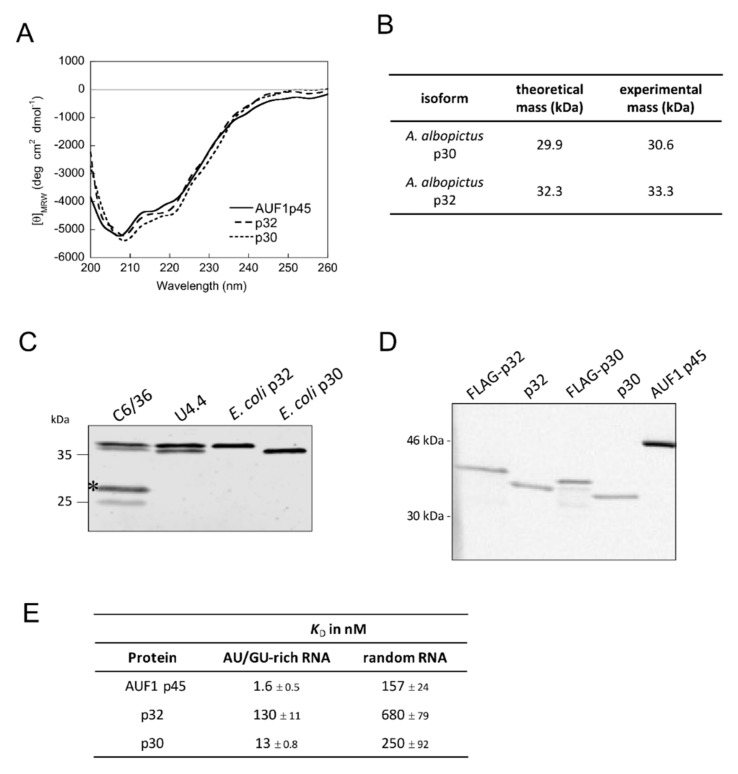
Characterization of mosquito proteins p30 and p32. (**A**) Far-UV circular dichroism (CD) spectra of AUF1 p45 and mosquito p30 and p32 were recorded. The acquired data were normalized to mean residue weight (MRW) ellipticities. The CD data for AUF1 p45 were taken from [16]. (**B**) Summary of the analytical ultracentrifugation experiments demonstrating that mosquito proteins p30 and p32 are monomeric proteins (see Supplementary Figure S3). (**C**) Detection of squid isoforms p30 and p32 in cell extracts of mosquito cell lines C6/36 and U4.4. An antibody that was raised against the full-length p32 protein (purified from *E. coli*) was applied. The asterisk indicates bands that most likely correspond to degradation products of p30. (**D**) In vitro methylation assay with PRMT1 and different protein preparations. Equal amounts (1 pmol) of mosquito proteins p30 and p32 that were purified from *E. coli,* as well as FLAG-p30 and FLAG-p32 proteins that were purified from C6/36 cells, were methylated by PRMT1. AUF1 p45 (13 pmol) that was purified from *E. coli* served as a positive control. The samples were taken after 2 h and analyzed by SDS–PAGE and phosphor imaging. (**E**) RNA binding affinities of human AUF1 p45 and mosquito proteins p30 and p32 to an AU/GU-rich RNA and a randomly composed RNA. Dissociation constants and standard deviations derived from at least three measurements. The binding data for AUF1 p45 were taken from [16].

Next, it was important to understand whether the two mosquito proteins squid p30 and p32 showed comparable or different activities as compared with the human AUF1 p45. In order to characterize the two isoforms in terms of RNA binding and RNA remodeling, the proteins were heterologously produced in *E. coli* and purified to homogeneity (Figure 5C). The far-UV CD spectra of squid p30 and p32 closely resembled the one of AUF1 p45. The low CD signal emphasized the high content of disordered regions, particularly at the N- and C-termini (Figure 6A). Analytical ultracentrifugation demonstrated a monomeric state of squid p30 and p32, which was previously shown to be the case for AUF1 p45 as well (Figure 6B and Supplementary Figure S3) [14].

Staining of cell extracts derived from two mosquito cell lines (C6/36 and U4.4) with an antiserum raised against the full-length p32 protein revealed two proteins migrating at the same position as the proteins squid p30 and p32 purified from *E. coli*, indicating that these are the only isoforms generated by the two gene loci (Figure 6C). It has been previously shown that AUF1 p45 is post-translationally modified by arginine methylation in human cells and that a methylated variant shows a considerably higher RNA chaperone activity [14]. To test if the squid p30 and p32 proteins are methylated in mosquito cells, we performed an in vitro methylation assay with immuno-affinity purified FLAG-tagged p30 and p32 from mosquito cells (C6/36) as compared with the respective proteins purified from *E. coli*. All proteins were equally well methylated by a methyltransferase, which indicated that, in contrast to AUF1 p45 in human cells, squid p30 and p32 are not methylated in mosquito cells (Figure 6D).

### 3.6. Mosquito Proteins p30 and p32 Show Activities Similar to AUF1 p45

Since AUF1 shows specificity for AU/GU-rich sequences, we sought to analyze the binding efficiency of squid p30 and p32 to a 16 nt long AU/GU-rich, single-stranded RNA and to a randomly composed single-stranded RNA of the same length. We conducted fluorescence equilibrium measurements using short, fluorescently-labeled RNA molecules. Binding of the AU/GU-rich RNA by squid p30 (*K*_D_ = 13 nM) and squid p32 (*K*_D_ = 130 nM) was found to be eight-fold and 80-fold less efficient than with AUF1 p45, respectively. Binding of the random RNA by squid p30 (*K*_D_ = 250 nM) and squid p32 (*K*_D_ = 630 nM) was 1.6-fold and four-fold less efficient than with AUF1 p45, respectively (Figure 6E). When comparing both RNA substrates, both squid isoforms displayed a higher affinity for the AU/GU-rich RNA than for the random RNA (19-fold for squid p30 and five-fold for squid p32). The *A. albopictus* squid isoforms p30 and p32, therefore, show a substrate preference for AU/GU-rich RNA similar to AUF1 p45. However, the RNA binding efficiency and substrate preference for AU/GU-rich sequences were not as pronounced as was the case with AUF1 p45.

**Figure 7:**
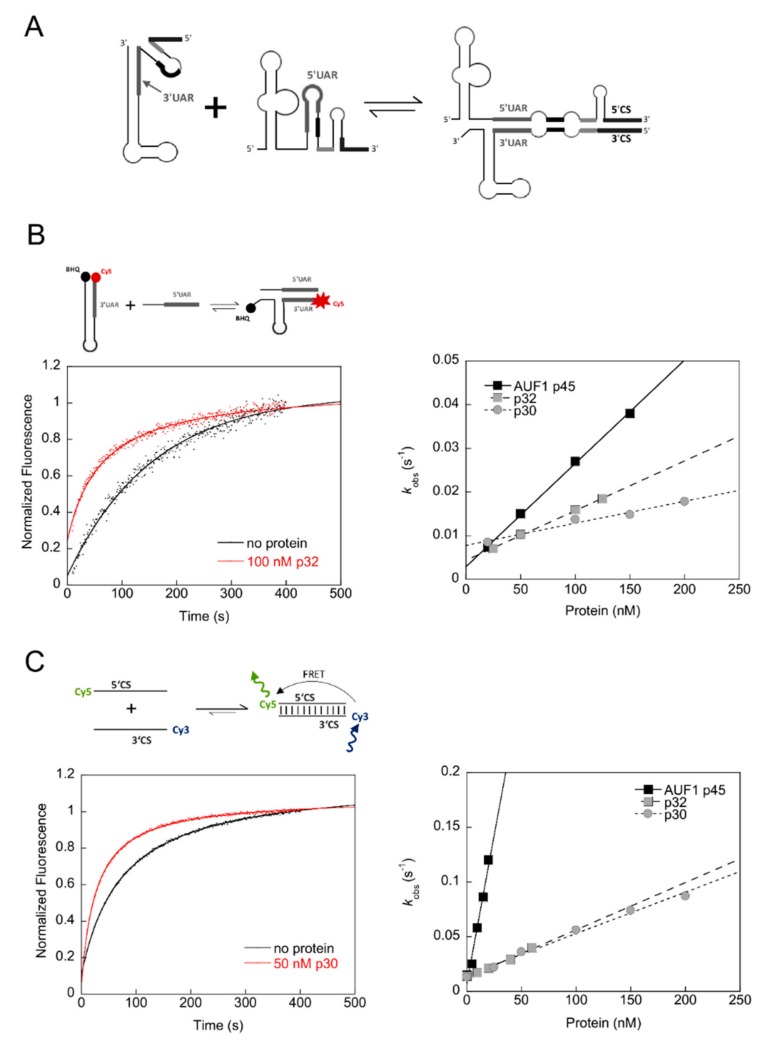
Mosquito proteins p30 and p32 exhibit RNA chaperone and RNA annealing activities. (**A**) Scheme of the structural rearrangement of the 5ʹ and 3ʹ termini, specifically of the 5ʹUAR and 3ʹUAR, as well as 5′CS and 3′CS elements, during cyclization of the WNV RNA genome. (**B**) (Top) Scheme of the fluorescence-based 3′SL^trunc^-5ʹUAR chaperone assay to detect protein-mediated conformational rearrangement of WNV RNA by dequenching of Cy5. (Bottom) Exemplary kinetic traces with Cy5 and BHQ (black hole quencher) labeled 3′SL^trunc^ incubated with or without 100 nM of p32. Following the addition of 5ʹUAR RNA the fluorescence signals were measured, plotted as a function of time, and fitted according to a first-order reaction (no protein, Equation (2)) or second-order reaction (in the presence of protein, Equation (3)). (Right) The observed rate constants *k*_obs_ (s^−1^) that were measured for the RNA chaperoning reaction in the presence of AUF1 p45, p30, or p32 were plotted as a function of the protein concentration. (**C**) (Top) Scheme of the fluorescence-based 5ʹCS-3ʹCS RNA annealing assay to analyze the hybridization of the CS cyclization sequences. Annealing of the complementary 5′- and 3′CS RNAs that are fluorescently labeled with Cy5 and Cy3, respectively, leads to a detectable FRET signal. (Bottom) Exemplary kinetic traces of the RNA–RNA interaction in the absence or presence of 50 nM p30. The fluorescence signals were analyzed according to a second-order reaction (Equation (3)). (Right) The observed rate constants *k*_obs_ (s^−1^) that were measured for the RNA annealing reaction in the presence of AUF1 p45, p30, or p32 were plotted as a function of the protein concentration.

In the following set of experiments, we applied two assays to evaluate the RNA remodeling capabilities of the *A. albopictus* squid isoforms p30 and p32. First, we applied an established fluorescence-based assay to mimic the rearrangement of the WNV RNA’s 3′-terminal SL (Figure 7A). To enable genome cyclization, the 3′SL has to be denatured to allow 5′-3′UAR hybridization (schematically depicted in Figure 7A). Using this assay, we previously demonstrated that AUF1 p45 exhibits an RNA chaperone activity, which promotes the destabilization of flaviviral stem-loops involved in genome cyclization [13,15]. The assay applies a Cy5- and black-hole quencher (BHQ)-labeled RNA oligonucleotide (3′SL^trunc^) corresponding to the lower part of the WNV 3′SL, including the 3′UAR sequence. The second RNA is single-stranded and corresponds to a sequence of the WNV 5′UTR mainly comprising the 5′UAR cyclization sequence. Hybridization of both RNAs via the UAR elements requires a rearrangement of the SL, which dislocates the Cy5 fluorophore from the BHQ and can be measured time-dependently (exemplary kinetic traces shown in Figure 7B). The rate constants were plotted against the protein concentration and revealed that the mosquito proteins displayed RNA chaperone activities. However, these were less efficient in supporting the 3′SL^trunc^–5′UAR interaction as compared with AUF1 p45, with squid p32 being more active than squid p30 (Figure 7B). Interestingly, there is no correlation between RNA binding and RNA chaperoning, with p30 displaying stronger RNA binding but lower RNA chaperoning activity as compared with p32. A similar situation was observed with the RNA chaperone StpA where strong RNA binding led to RNA stabilization and was found to be detrimental to RNA chaperone activity [18].

In a second approach, we investigated whether the mosquito squid proteins could promote the annealing of single-stranded complementary RNAs. For this purpose, we applied a previously established FRET-based RNA annealing assay using the conserved flaviviral 5′ and 3′CS cyclization sequences, which are labeled with a Cy5 and a Cy3 fluorophore, respectively (exemplary kinetic traces shown in Figure 7C) [14]. The rate constants revealed that the mosquito squid proteins displayed a considerably lower RNA annealing activity than that of AUF1 p45 (Figure 7C).

These data show that the mosquito squid proteins share RNA remodeling activities similar to AUF1 p45, however, both the RNA chaperone and especially the RNA annealing activities of p30 and p32 are less efficient as compared with AUF1 p45.

### 3.7. The RNA Chaperone Activity of AUF1 p45 is Enhanced at Higher Temperature

**Figure 8:**
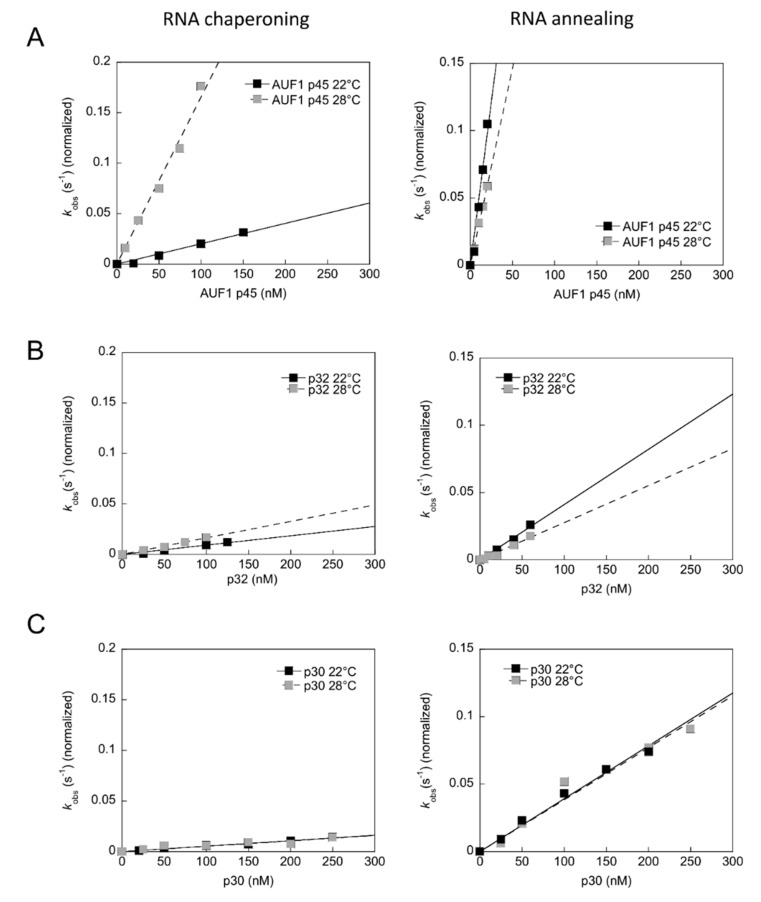
The effect of temperature on rates of RNA chaperoning and RNA annealing reactions. The RNA chaperoning (left) and RNA annealing (right) reactions in the presence of AUF1 p45 (**A**), p32 (**B**), or p30 (**C**) were performed at 22 °C and 28 °C. The rate constants were normalized by subtracting the non-enzymatic rate from the total rate and plotted as a function of the protein concentration.

Since the temperature differentially affected the replication efficiency of WNV reporter replicon RNAs in human and mosquito cells, we wondered if the RNA remodeling activities of the human AUF1 p45 and the mosquito p30 and p32 were also temperature dependent. Therefore, we repeated the RNA remodeling assay, which is normally conducted at 22 °C, at 28 °C. Interestingly, we observed that the RNA chaperone activity of AUF1 p45 was strongly enhanced at 28 °C, whereas the RNA chaperone activities of p30 and p32 were not, or were only mildly, improved at higher temperature, respectively (Figure 8A–C). The RNA annealing activity of p30 was not affected by the temperature shift, while p32 and AUF1 p45 showed slightly lower RNA annealing activities at the higher temperature (Figure 8A–C). These data revealed important differences in the properties of the mosquito and human proteins at different temperatures.

## 4. Discussion

In order to maintain their natural transmission cycle, arboviruses are required to establish persistent non-lethal infections in the mosquito host that do not significantly interfere with the vector’s physiology, and therefore transmission efficiency. However, the relationship between vector-borne viruses and their arthropod vectors need not be benign, with the magnitude of virulence depending on the vector and virus taxonomic groups and the mode of transmission [19]. Evolutionary conserved pathways such as the Jak-STAT and Toll pathways (reviewed in [20]) as well as the RNAi pathway representing the major antiviral response have been shown to limit the replication of arboviruses in mosquitos [21,22,23]. As a countermeasure, flaviviruses produce a non-coding viral RNA called sfRNA, which acts as a suppressor of the RNAi response, and hence is a key driver to overcome the mosquito midgut infection barrier [24,25]. Temperature is another critical factor since the insect vectors are ectothermic and susceptible to temperature changes. Higher temperatures have been associated with increased infection rates and a decreased extrinsic incubation period in mosquitoes infected with WNV [26,27,28,29]. However, the role of temperature in virus transmission is controversially discussed, and the molecular mechanisms underlying the effects of temperature on virus replication efficiency, and therefore vector competence are largely unclear [30]. Adelman and colleagues have previously demonstrated the importance of temperature on mosquito physiology by showing that cooler rearing temperatures destabilize RNAi and increase susceptibility of mosquitoes to viral infection [31].

During the natural process of host switching, invertebrate and vertebrate species impose different selective forces on flaviviral populations with differential impacts on viral mutational diversity and relative fitness [32,33,34,35,36,37,38]. In addition to host-specific requirements, one factor that is usually not taken into consideration is the temperature shift that flaviviruses undergo during host switching. Interestingly, the structure of the DENV virion shows a remarkable adaptation to the different temperatures of the human and mosquito hosts [39,40]. In this study we propose a model in which the temperature has a direct influence on local RNA structure, and therefore on long-range RNA–RNA interactions relevant for WNV replication. We provide evidence that not only the conformational flexibility of the conserved RNA structure 3′SL, but also the temperature is a critical determinant of replication kinetics.

**Figure 9:**
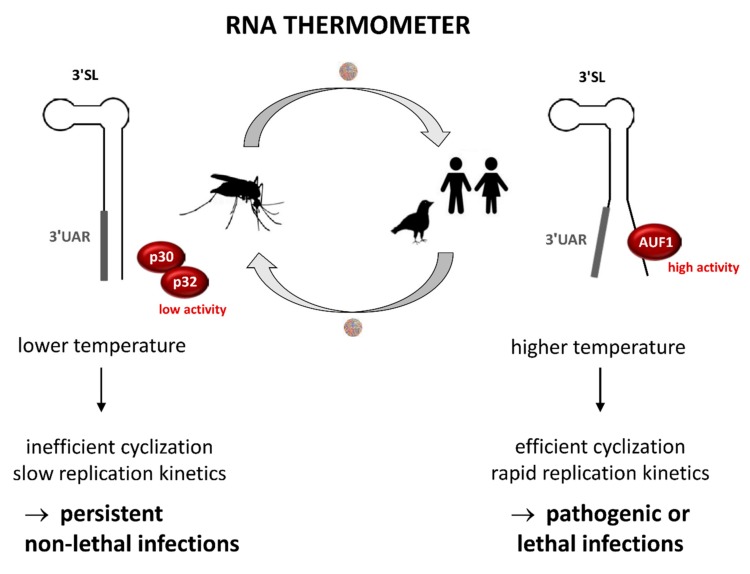
A model of the WNV 3′SL acting as an RNA thermometer during host switching. Due to the lower body temperature and low activities of p30/p32 in the mosquito host, the 3′SL exhibits a higher stability, which leads to inefficient cyclization and slow replication kinetics. In this way, persistent non-lethal infections in mosquitos can be established. In vertebrate hosts the higher body temperature and the strong activity of AUF1 renders the 3′SL more flexible. Consequently, cyclization is efficient, replication is fast, and high viral titers are produced, which can lead to pathogenic or lethal infections.

In mosquito and human cells, we observed slower WNV replicon replication kinetics at 28 °C and faster replication kinetics at 37 °C, similar to a study that was done with infectious WNV [41]. Flavivirus 5′-3′-genome cyclization through long-range RNA–RNA interactions is essential for RNA replication. Opening of the 3′SL, which contains the 3′UAR cyclization sequence, is required for 5′-3′-genome cyclization and initiation of RNA synthesis by NS5. In this regard, a silencing effect of the 3′SL in the context of RNA synthesis was previously proposed for DENV [12]. We have shown that WNV 5′-3′ RNA-RNA interaction in vitro is enhanced at higher temperatures (Supplementary Figure S4). Therefore, it is reasonable to assume that at lower temperatures, such as in the mosquito host, cyclization of the genome is less efficient, and therefore RNA replication starts at later time points. Such temperature constraints on virus replication within the mosquito, which is mediated by a silencing effect of the 3′SL, could be beneficial for the virus and could serve two purposes which include: (1) Low virus titers help to ”fly under the radar” of the RNAi immune response; and (2) cytopathic effects are avoided, therefore, preventing lethal flaviviral infection in mosquitos. Therefore, the increased stability of the 3′SL at lower temperature maintains the persistent nonpathogenic character of flavivirus infections, and in this way may facilitate flavivirus transmission in nature (see model in Figure 9).

The temperature shift, upon entering the natural vertebrate host, releases the silencing effect of the 3′SL leading to increased cyclization and replication rates and high virus titers accompanied by cytopathic effects. A so-called RNA thermometer activity in order to switch from a stable to a flexible 3′SL could be involved in modulating the replication efficiency during host switching (Figure 9). RNA thermometers are well characterized in bacterial pathogens where they coordinate temperature-dependent gene expression. These temperature-sensing structured RNA sequences switch from closed to open conformations and modulate translation efficiency [42].

With increasing temperature, the structural flexibility, and with this accessibility of the 3′SL is believed to increase, which not only facilitates cyclization of the flaviviral genome, but is also likely to stimulate the activity of cellular host factors that modulate RNA conformations. In this study we demonstrate that a defined conformation, and, with this, a high structural flexibility of the 3′SL, enhanced the RNA chaperone activity of the flavivirus host factor AUF1 p45, which, in turn, favored RNA synthesis of WNV (Figure 2).

We identified AUF1-homologous proteins in mosquito cells that are capable of remodeling viral RNA structures; however, it is not clear if squid p30 and p32 act as host factors for flavivirus replication in mosquitos similar to the situation with AUF1 p45 in human cells. Our attempts to identify a function of p30 and p32 during WNV replication in mosquito cells by knockdown or knockout experiments did not reveal a supportive role [43]. There could be several explanations as to why AUF1 p45 promotes WNV replication in human cells [13] but p30 and p32 in mosquito cells do not as follows: (1) p30 and p32 show RNA remodeling activities, however, these are less efficient as compared with AUF1 p45′s, and in particular the RNA annealing activity of the mosquito proteins is strikingly lower than that of AUF1 p45 (Figure 7C); (2) p30 and p32 in mosquito cells were not found to be methylated (Figure 6D), a post-translational modification that turned the human AUF1 p45 into a much stronger RNA chaperone [14]; (3) the RNA chaperone activity of AUF1 p45 is much stronger at higher temperature, whereas the RNA chaperone activities of p30 and p32 are not, or only slightly better, respectively (Figure 8); (4) there are altogether four AUF1 isoforms in human cells [44], the activities of which in terms of RNA remodeling are currently under investigation [45]. The presence of four isoforms in human cells as compared with only two isoforms in mosquito cells might be beneficial. Therefore, based on these findings it is conceivable that the role as a host factor evolved later on in vertebrates, where the proteins are methylated, show higher RNA remodeling activities, and a stronger tendency to be more active at higher temperatures.

In addition to AUF1, there might be other host RNA-binding proteins that modulate the structure of the 3′SL and which are involved in the flaviviral RNA switch from linear to circular. Such a role was previously proposed for eEF1A which binds to the 3′SL [11,46,47]; however, 3′SL restructuring by eEF1A still needs to be experimentally confirmed.

In summary, we propose a molecular mechanism describing a constellation where temperature, a viral RNA structure element, and a host factor activity are functionally connected. It has been suggested that a fine balance between the linear and the circular conformation is crucial for flavivirus replication [48] and that genome cyclization can even inhibit translation initiation on ZIKV and DENV RNA [49]. The RNA thermometer activity of the flaviviral 3′SL described here adds an additional layer of regulation to the switch from translation to replication in a time- and temperature-specific manner. The temperature-adaptable folding of the 3′SL could, therefore, be a decisive signal for the viral pathogen in order to regulate the rate of replication during host switching and could be crucial in maintaining the mosquito-vertebrate transmission cycle.

## Supplementary Materials

The following are available online at https://www.mdpi.com/1999-4915/12/1/104/s1, Figure S1: Time course of Rluc activity in cells transfected with WNV replicons, Figure S2: Exon-intron organization of two genomic loci in C6/36 cells encoding AUF1-homologous proteins, Figure S3: Mosquito squid p30 and p32 are monomeric proteins, Figure S4: 5’-3′ RNA-RNA interaction is increased at higher temperatures, Table S1: DNA oligonucleotides used in this study, Table S2: RNA oligonucleotides used in this study.
