# Supplementary material for: An RNA Thermometer Activity of the West Nile Virus Genomic 3′-Terminal Stem-Loop Element Modulates Viral Replication Efficiency during Host Switching
Source: Viruses. 2020 Jan 15;12(1):104. doi: 10.3390/v12010104 (PMC7019923; doi:10.3390/v12010104)
Supplement: Supplementary file 1 [file viruses-12-00104-s001.pdf]

Supplementary Table 1

DNA oligonucleotides used in this study

name	sequence
**cloning of cDNAs encoding different WNV replicon RNA**	
3SLMut1For	GGTGCGAGAACAGAGGATCTGGGTCG
3SLMut1Rev	CGACCCAGATCCTCTGTTCTCGCACC
3SLMut2For	GGTGGTGCGAGAGCACAGGATCTGG
3SLMut2Rev	CCAGATCCTGTGCTCTCGCACCACC
3SLMut3For	GGTGGTGCGAGAGCAGAGGATCTGGGTCG
3SLMut3Rev	CGACCCAGATCCTCTGCTCTCGCACCACC
**PCR templates for in vitro transcription**	
5UTRWestFor	ATAAGCTTTAATACGACTCACTATAGGAGTAGTTCGCCTGTGTGAGCTGA
3UTRnativRev	AGATCCTGTGTTCTCGCACCACCAGCC
3'UTRRevMut1	AGATCCTCTGTTCTCGCACCACC
3'UTRRevMut2	AGATCCTGTGCTCTCGCACCACC
3'UTRRevMut3	AGATCCTCTGCTCTCGCACCACC
**Exon-intron PCR**	
C636 Exon4 Fw	AAGGAGGTGGACGTCAAGAAGG
C636 Intron4 Rv	GCTTCAGTTTTCAAGCACTGAAGG
**SUMO expression plasmids**	
AUF1 Aedes BsaIFor	ATGGTCTCATGGTGCCGATCAGGATCAAGAG
AUF1 Aedes BamHIRev	ACGGATCCTTAGTACGGCGTATGCCTTGG
**FLAG-p30 and p32 fusion transcribed from pSinRep5**	
C636AUF1XbaFw	ATTCTAGAGCCACCATGGATTACAAGGATGACGACGATAAGGCCCGG GCGGATGCCGATCAGGATCAAGAGATG
C636AUF1ApaIRv	ATGGGCCCTTAGTACGGCGTATGCCTTGGCTG
**cloning of cDNAs encoding WNV replicon NS5mut RNA**	
NS5PolQuikFor	GCTGTCAGTGGAGATGCCTGTGTGGTAAAGCCC
NS5PolQuikRev	GGGCTTTACCACACAGGCATCTCCACTGACAGC

Supplementary Table 2

RNA oligonucleotides used in this study

**name**	**sequence**
3'SL^{trunc}	Cy5-AGAUCUUCUGCUCUGCACAAUGGUGCGAGAACACAGGAUCU-BHQ
5'UAR	UCUUAGCACGAAGAUCU
AU/GU-rich RNA	FAM-EX-5'-UAUUUAGUGGUGUUAG-3'
random RNA	FAM-EX-5'-CUAAGAUGCUCGCUGC-3'
5CS WNV Cy5	Cy5-UGUCAAUAUGCUAA
3CS WNV Cy3	Cy3-AGCAUAUUGACACC

Supplementary methods

Plasmid encoding WNV NS5mut replicon

To obtain replicon-encoding cDNAs with mutations in the NS5 gene, the SpeI-SacII fragment of pWNV (Shi et al., 2002) was subloned into pGEM-T Easy. Site-directed mutagenesis was carried out using primers NS5PolQuikFor and NS5PolQuikRev. The SpeI-SacII fragment with the corresponding mutation was cloned into pWNVRluc.

RNA gel-shift assay

RNA-RNA interactions were analysed by electrophoretic mobility shift assays as performed earlier (Friedrich et al., 2014). The binding reaction contained 5 mM Hepes/NaOH, pH 7.9, 100 mM KCl, 5 mM $MgCl_2$, 1 mM dithiothreitol, uniformly ^{32}P-labelled 5'-RNA (5' 162 Nt) (2 nM) and non-labelled 3'-RNA (3' 111 Nt) (2 nM) in a final volume of 20 µl. The binding reaction was performed at the indicated temperatures for 2 h. The RNA-RNA complexes were resolved by native 5% polyacrylamide gel electrophoresis in 1 x TBE at 4°C, analysed by phosphor imaging and quantified by ImageQuant Software (GE). Signal intensities were blotted as a function of 3'-RNA concentration and fitted by KaleidaGraph (Synergy) to a single-site binding model to determine the dissociation constant K_D:

$$S = S_{max} \bullet x / (K_D + x)$$

S - radioactive signal; S_{max} - maximal signal amplitude; x - concentration of the 3'-RNA; K_D - dissociation constant.

Supplementary Figures

Figure S1

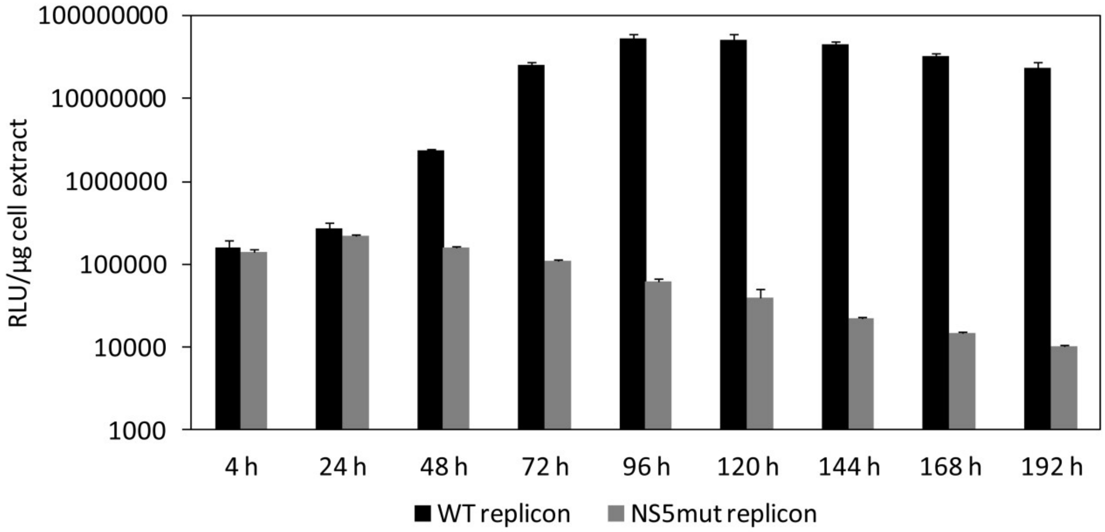

Fig. S1. Time course of Rluc activity in cells transfected with WNV replicons. Huh7 cells were transfected with wild-type and NS5mut WNVRluc replicon RNAs, subsequently cultivated at 28°C and analysed for luciferase reporter activity at the indicated time-points post transfection. Results from two independent transfections with two technical replicates are shown; error bars reflect standard deviations. Please note that the values for the Rluc activity are higher compared to results from the main manuscript due to a different luminometer used.

Figure S2

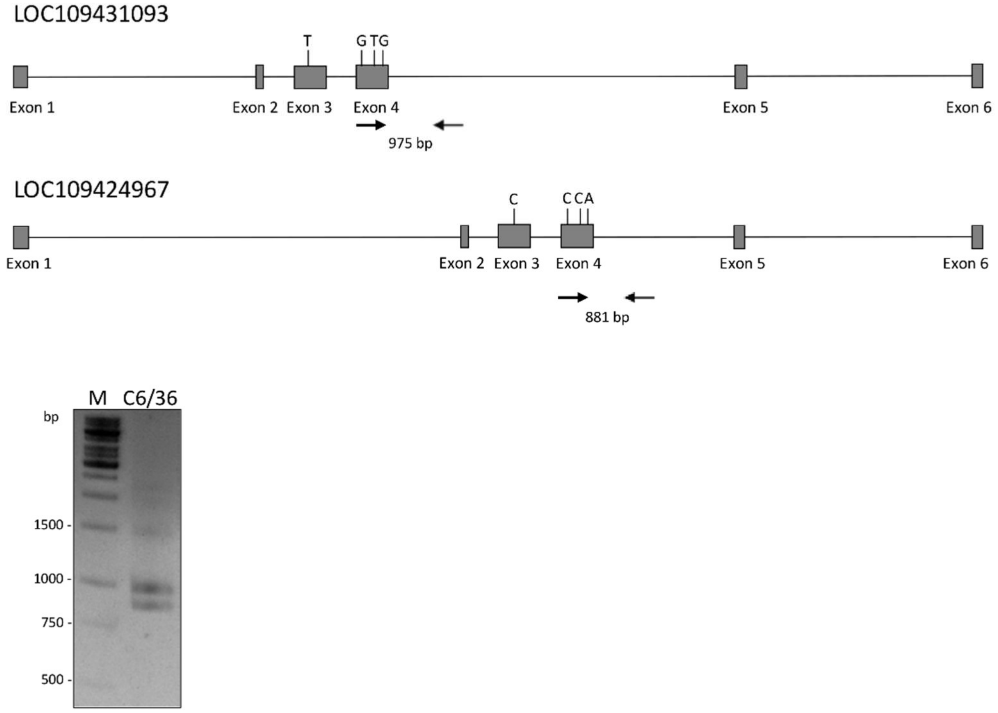

Fig. S2. Exon-intron organization of two genomic loci in C6/36 cells encoding AUF1-homologous proteins. (Top) The two loci mainly differ in their intron sequence. Nucleotide differences in exons 3 and 4 do not change the amino acid sequence. Arrows indicate the location of PCR primer binding sites. PCR product sizes are indicated. (Bottom) The PCR products from the exon-intron PCR were analysed on an agarose gel in parallel with a molecular weight marker (M).

Figure S3

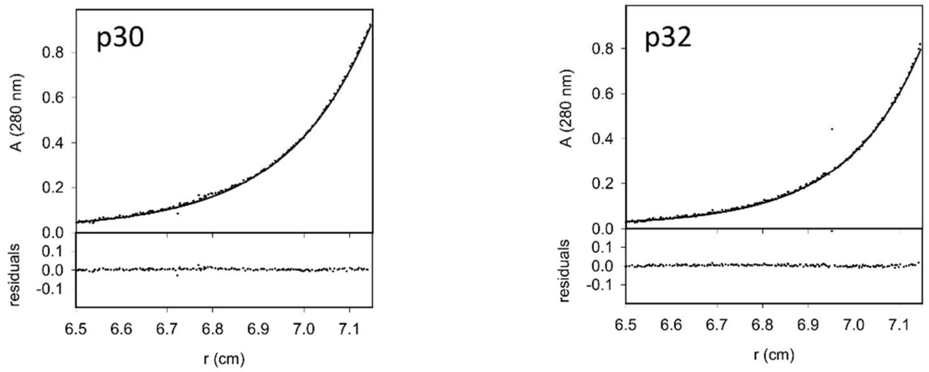

Fig. S3. Mosquito squid p30 and p32 are monomeric proteins. In sedimentation equilibrium experiments (analytical ultracentrifugation) 12 µM (p30) and 7 µM (p32) of protein was analyzed at 14000 rpm at 20°C. No aggregation of the proteins was observed in the course of the experiment.

Figure S4

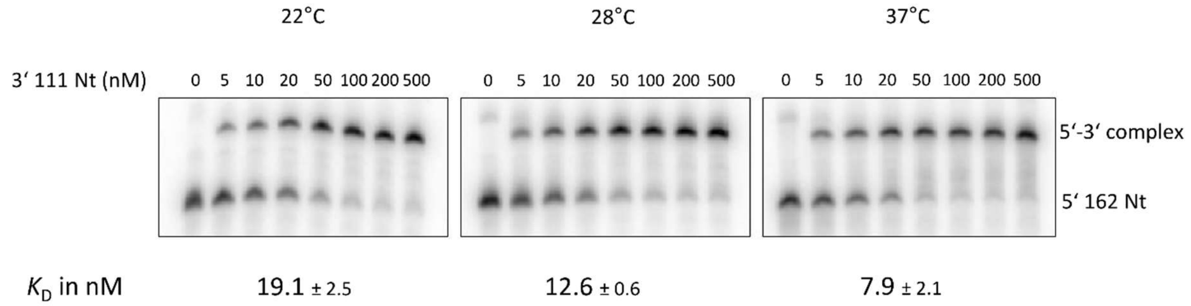

Fig. S4. 5’-3’ RNA-RNA interaction is increased at higher temperatures. ^{32}P-labelled 5’-RNA (5’ 162 Nt) (2 nM) and non-labelled 3’-RNA (3’ 111 Nt) (2 nM) were incubated at the indicated temperatures for 2 h. The RNA-RNA complexes were resolved by native 5% polyacrylamide gel electrophoresis, analysed by phosphor imaging and quantified by ImageQuant Software (GE). The signal intensities were fitted as a function of 3’-RNA concentration to obtain the binding affinities (shown below). Average results and standard deviations (n = 3) are shown.

References

1. Friedrich, S.; Schmidt, T.; Geissler, R.; Lilie, H.; Chabierski, S.; Ulbert, S.; Liebert, U.G.; Golbik, R.P.; Behrens, S.E. AUF1 p45 promotes West Nile virus replication by an RNA chaperone activity that supports cyclization of the viral genome. J Virol 2014, 88, 11586-11599, doi:JVI.01283-14
2. Shi, P.Y.; Tilgner, M.; Lo, M.K. Construction and characterization of subgenomic replicons of New York strain of West Nile virus. Virology 2002, 296, 219-233
